# Bilingual and Bicultural Research Teams: Unpacking the Complexities

**DOI:** 10.1089/heq.2019.0111

**Published:** 2020-06-09

**Authors:** Maya I. Ragavan, John D. Cowden

**Affiliations:** ^1^ Division of General Academic Pediatrics, University of Pittsburgh/Children's Hospital of Pittsburgh, Pittsburgh, Pennsylvania, USA.; ^2^ Division of General Academic Pediatrics, Children's Mercy Kansas City, Kansas City, Missouri, USA.

**Keywords:** bilingual, bicultural, academic medicine, health disparities

## Abstract

Researchers often describe use of “bilingual/bicultural” research teams, especially for research being conducted with marginalized communities. In this perspectives article, we argue that while increasing the diversity of research teams is imperative, using the term “bilingual/bicultural” without further explanation is problematic. We first review the nuances of the terms bilingual and bicultural individually. Next, we describe how the terms bilingual and bicultural cannot be conflated. Finally, we provide recommendations for researchers, journals, and funding agencies.

## Introduction

Over the past two decades, there has been a transformative paradigm shift in research to recruit and retain participants of diverse racial, ethnic, linguistic, and cultural backgrounds. In fact, a priority of the National Academy of Medicine is ensuring that participants in clinical trials reflect the overall diversity of the United States.^1^ A commonly suggested strategy is to include “bilingual and bicultural” research staff, with the premise being that participants may be more likely to join and remain involved in the research if research staff share their language and cultural identity.^2–4^ For example, researchers noted how using bilingual and bicultural staff may facilitate retention in a large cohort study of Hispanic/Latino participants.^5^ We agree that recruiting research participants of diverse backgrounds is imperative to ensure that emerging interventions and treatments apply to an increasingly diverse U.S. population. However, we argue that using the term “bilingual/bicultural” without adequate explanation is problematic and merits further exploration. Please note that when we use the term research staff, we mean all individuals involved in research, including the principal investigator, research assistants, community advisors, and other members of the research team. This article was deemed nonhuman subjects by our institutions' Institutional Review Boards.

## Complexities of the Terms Bilingual and Bicultural

The terms bilingual and bicultural must be separated and considered individually. Bilingual is defined as having some degree of proficiency in two languages. Although bilingualism often is treated as a binary concept (i.e., a person is either bilingual or not), language proficiency lies on a spectrum and is specific to a context.^6^ Because of this, bilingualism in health care settings has begun to take on a professional definition as the importance for specific levels of proficiency depending on context has become clear.^7–9^ In the past, any degree of bilingual ability was considered “better than nothing” in medical interactions, and self-assessment of language competency was allowed and even encouraged. Recognizing the threat this poses to the quality and safety of care, health care centers have begun requiring clinicians and research staff to prove their language ability through validated testing before being allowed to give care or interact with research participants. This mirrors the requirements of many institutional review boards that translation of consent forms, surveys, and recruitment materials be conducted by a certified translator. Nevertheless, there are still places where research team members might simply state that they are bilingual and be allowed to work in a non-English language. Future research will need to assess how to best teach, test, and maintain language abilities for research staff.

The term bicultural is even more challenging to define. Broadly speaking, a person is bicultural if he or she identifies with more than one cultural heritage. However, there is considerable variability in what this could mean. When someone immigrates to the United States, he or she may begin adopting U.S. cultural practices, values, and identities, a process known as acculturation.^10^ First-generation immigrants (i.e., born abroad) and their second-generation children (i.e., born in the United States, at least one parent born abroad) may also hold on to certain cultural practices, identities, and values of their country of origin in different ways.^10^ Like language proficiency, acculturation is a dynamic rather than static process and an individual's level of acculturation may rapidly change depending on internal and external circumstances. Therefore, two people identifying as bicultural may share very different cultural values and lived experiences.

Within specific “cultural” groups, there may be considerable heterogeneity. Recognition of the socioeconomic, linguistic, and cultural differences among subethnic backgrounds (e.g., Vietnamese, Cambodian, and Indian, all part of “Asian” culture) has led to calls for disaggregation of traditional racial and ethnic groups used in the United States to better understand health disparities.^11,12^ Even when two people share a similar cultural heritage, they may have different lived experiences based on the context in which they are living. An immigrant who settles in an established ethnic enclave will likely have different experiences, access to resources, and acculturation patterns than the one who settles in a neighborhood with few people of a similar background.^10,13^ Social identities also can shift as bicultural individuals move between contexts, with terms used to differentiate people in the country of origin (e.g., ladino or indígena in Mexico) being replaced by new identities in the settlement country (e.g., Hispanic or non-Hispanic in the United States). Finally, cultural identity encompasses more than racial and ethnic background or country of origin, including socioeconomic status, gender identity, occupation, geographic location, and many other characteristics. Someone who has lived in both urban and rural communities or grew up in poverty, but then became part of the middle class, may consider himself or herself bicultural.

Pediatric research, especially when it includes parent and child participants, is further complicated by the fact that each member of a family may acculturate differently. Immigrant children, especially those who were born in the United States or who moved to the United States at a young age (1.5-generation immigrants), may acculturate differently than their parents.^10,14,15^ Therefore, even within a specific family, individuals may hold disparate cultural values, identities, and language preferences, and so, what may be culturally appropriate for a parent may be different than what is culturally appropriate for a child. Acculturation differences may also impact cultural alignment between participants and research team members. For example, in a study with immigrant parents and their adolescent children, bicultural research staff who moved to the United States at a young age may identify more with the culture of the adolescent rather than the parent.

Research staff who identify as bicultural may benefit from having insider knowledge about a community, but may also come with unique perspectives based on their lived experiences, where they were born, and how they identify.^16^ Understanding these perspectives is critical to determine how bicultural research staff engage with participants. This may be particularly important for research where reflexivity (e.g., the effect of the researcher's role) is critical to data collection, such as with qualitative research.^17,18^ For example, I (M.R.) have worked with research assistants who identify as bicultural and, due to understanding a culturally salient comment a participant made about traditional medicine, did not query further during a semistructured interview. While the RA built a rapport with the participant, thus yielding a stronger interview, that insider knowledge kept them from gathering further information relevant to the research study. Similar observations were made in an article describing lessons learned from conducting community-engaged research with the Hmong community. The authors noted that while including a research investigator who identified as bicultural was vital to the project's success, it also required the investigator to be aware of how her position as a “cultural insider” impacted her interactions with participants.^16^

## Bilingual and Bicultural Should Not Be Conflated

The terms bilingual and bicultural should not be conflated. An individual may be able to fluently speak multiple languages without culturally identifying with the countries from which those languages originate. Conversely, someone may identify culturally with a certain region, but not comfortably speak the language. This may be particularly true for people identifying as second-generation or third-generation immigrants (e.g., one grandparent born abroad), who are less likely than first-generation immigrants to speak the language from their heritage country.^19^ The differences in the terms bicultural and bilingual reflect the lived experiences of the authors. MR, for example, is of Asian American heritage but was born and brought up in the United States. She has travelled extensively in her parent's country of origin and considers herself bicultural. However, she also speaks Spanish, although does not identify as Hispanic/Latina. JC, on the contrary, grew up in a racially homogeneous suburb in the Midwest but has lived and worked in Mexico intermittently for 25 years. He speaks fluent Spanish and has passed a validated test of health care language proficiency but does not identify as Hispanic/Latino or bicultural, regardless of his extensive contact with Mexican culture abroad and in the United States.

## Recommendations for Researchers, Journals, and Funding Agencies

While we applaud the use of research staff that reflects the growing diversity of the United States, we believe researchers, journals, and funding agencies must consider unpacking the term “bilingual/bicultural.” Researchers should leverage the expertise of community-based organizations who serve the populations that a researcher wishes to include. Community-based participatory research is a well-documented technique for eliciting community feedback about the research process, including which research staff are most appropriate to include in a particular study.^20,21^ When developing a research team, it may be important to ensure that individuals with multiple cultural identities and linguistic preferences are represented. One strategy is conducting facilitated sessions to provide research staff the opportunity to reflect on their cultural and linguistic identities and consider how those identities are relevant to the research study. These sessions should be conducted in a safe and open manner, with the understanding that not all members of a research team may wish to disclose their cultural identities. Training for research staff on how their bilingual and/or bicultural identities might affect their research activities is also important. We also support bolstering efforts to increase the diversity and inclusion in all members of research staff, including principal investigators, as recommended by the National Academy of Medicine.^1^ Funding agencies and journals may consider having researchers describe, in grants and articles, exactly how the research team was bilingual and/or bicultural, how that may strengthen or limit their research, and specific training provided to the research team, including assessments to verify linguistic abilities of bilingual team members. Funding agencies should also ensure that teams have adequate funding and resources to include community-based partners, who can advise on the type of bicultural and bilingual research staff most appropriate for the project.

## Conclusions

Inclusion of diverse research staff who reflect the identities of participants is critical to address health disparities and achieve health equity. A more nuanced understanding and disaggregation of the terms bilingual and bicultural will allow research teams to capitalize on community knowledge without making assumptions about individuals' identities. Respectful translation of community knowledge is the first step to ensuring equitable inclusion of diverse populations in health research.

## Disclaimer

The content is solely the responsibility of the authors and does not necessarily represent the official views of the National Institute of Health.
